# A prospective study on single‐port versus multiport patient‐reported surgical outcomes

**DOI:** 10.1002/bco2.290

**Published:** 2023-09-15

**Authors:** Luca A. Morgantini, Ahmad Alzein, Arthi Bharadwaj, Matthew S. del Pino, Erin Egan, Ashwin Ganesh, John Smith, Simone Crivellaro

**Affiliations:** ^1^ Department of Urology University of Illinois at Chicago Chicago Illinois USA

**Keywords:** multiport, patient reported outcomes, prospective study, single port, surgical scars

## Abstract

**Introduction:**

We sought to determine potential patient reported advantages of the da Vinci single‐port (SP) robotic system for urological procedures compared with the previous model, the da Vinci multiport (MP) system. The SP model utilizes a single 30 to 40 mm incision rather than multiple 5 to 22 mm incisions. This project aims to prospectively investigate the impact of the novel SP system on patient reported cosmetic and psychometric surgical outcomes.

**Methods:**

We conducted a prospective study of patients who underwent uro‐oncologic surgery by three urologists at the University of Illinois Chicago from April to November 2021. Study participants completed a Patient Scar Assessment Questionnaire 20 and 90 days post‐procedure. The Patient Scar Assessment Questionnaire is a reliable measure of surgical scars that includes five subscales: Appearance, Symptoms, Consciousness, Satisfaction with Appearance, and Satisfaction with Symptoms. Higher scores represented worse reported outcomes.

**Results:**

On Postoperative Day 20, there were 77 responses (53 SP and 24 MP). Patients receiving SP procedures reported more favourable outcomes in terms of appearance, symptoms, consciousness, and pain medication. On Day 90, there were 37 responses (24 SP and 13 MP). Patients receiving SP procedures reported more favourable outcomes in terms of appearance. No significant differences were seen on Day 90 in terms of pain, medication, symptoms, consciousness, or satisfaction.

**Conclusions:**

This study demonstrates the superiority of the SP in patient reported cosmetic and pain outcomes on short‐ and long‐term follow‐up after uro‐oncological surgical procedures. Symptomatic and cosmetic advantages are present at the 20 day follow‐up, with better scar appearance being significant 90 days after surgery.

## 1 INTRODUCTION

Since the 2018 FDA approval of the da Vinci single‐port (SP) robotic system for urologic surgery, the SP model has been quickly adopted among academicians and private practitioners as a safe and efficient alternative approach to the already widespread da Vinci multiport (MP) systems available: S, Si, and Xi models. Initial investigation and case reports of successful implementation of the SP system for complex urologic surgeries suggest favourable efficacy of this robot model compared with its predecessors.^1^, ^2^, ^3^, ^4^, ^5^ The SP approach uses a single surgical arm and 30 to 40 mm incision, as opposed to three to five incisions of 5 to 22 mm necessary for using MP models. The similarities between MP and SP systems allow for experienced surgeons to adapt to the SP model, while the need for only a single skin and fascial incision may allow for improved patient perception of surgery scars as well as reduced pain during recovery.^6^

In urology, especially, robotic surgery has rapidly become the standard approach for most minimally invasive cases^7^ In pure laparoscopic cases, there has been in the past an attempt towards laparo‐endoscopic single site surgery. Despite multiple investigations that report both improved perioperative and cosmetic outcomes compared with multiport laparoscopic approaches in urology,^8^, ^9^, ^10^ laparo‐endoscopic single site surgery never reached mainstream. We believe this is mostly related to the long learning curve and the limitations of the laparoscopic instruments in a single incision setting. Most of these limitations have been overcome by its robotic version.

The SP DaVinci is most often used within urology for radical prostatectomy, nephrectomy, and cystectomy, though it has wide potential for nearly every area of robotic urologic surgery, including complex reconstruction.^11^ Evidence from recent investigations report equal or improved intraoperative and surgical outcomes using the SP approach, such as equivalent operative time and complication rates with reduced blood loss compared with MP approaches.^12^, ^13^

Following an initial retrospective analysis comparing patient satisfaction with scarring after surgery with the SP versus MP da Vinci approach, we initiated a prospective study reporting patient interpretation of cosmetic outcomes and pain management in recovery from uro‐oncologic surgery using SP versus MP da Vinci approaches.

## 2 METHODS

**FIGURE 1:**
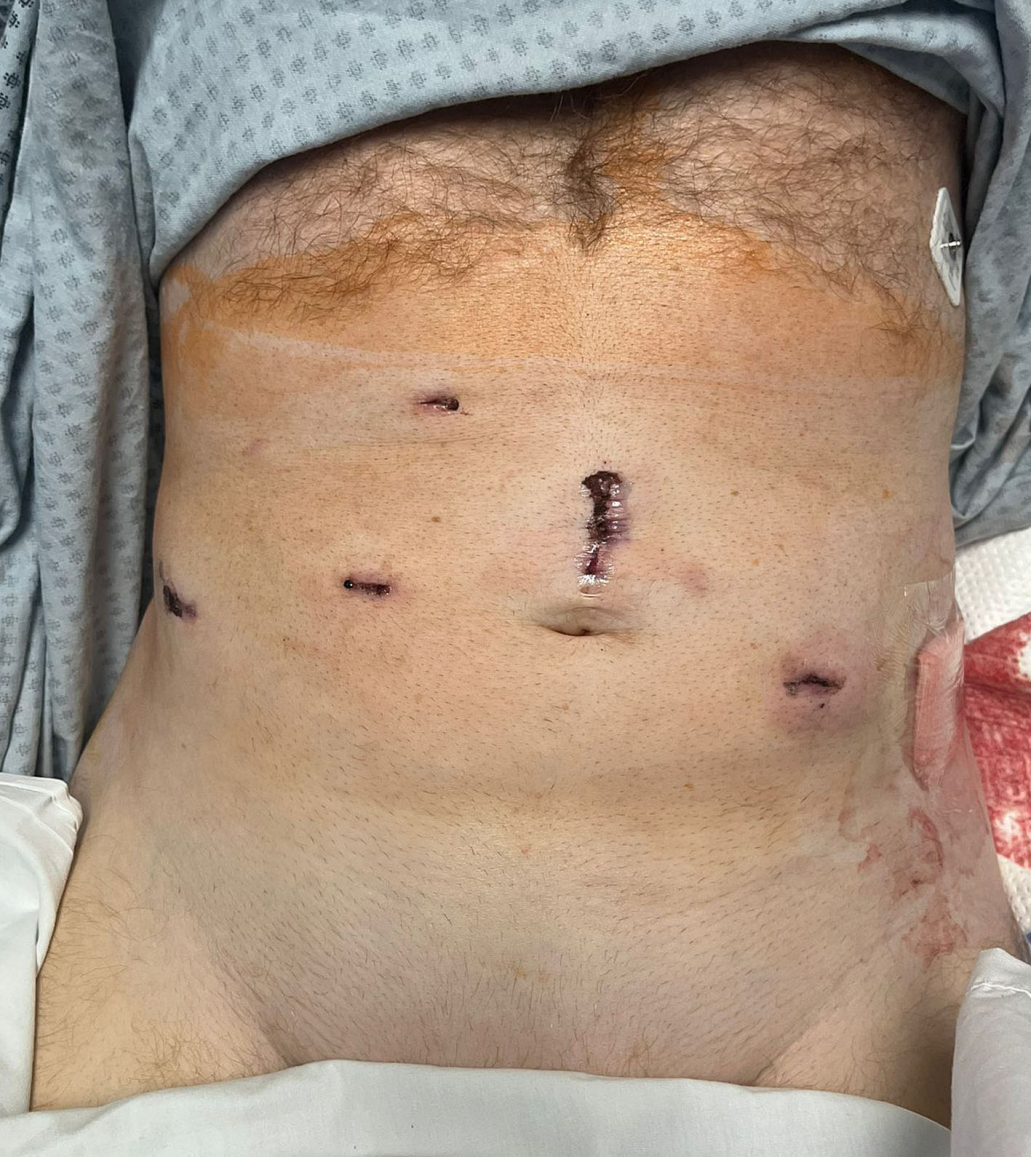
Sample multiport incisions, in this case Xi‐assisted robotic prostatectomy.

**FIGURE 2:**
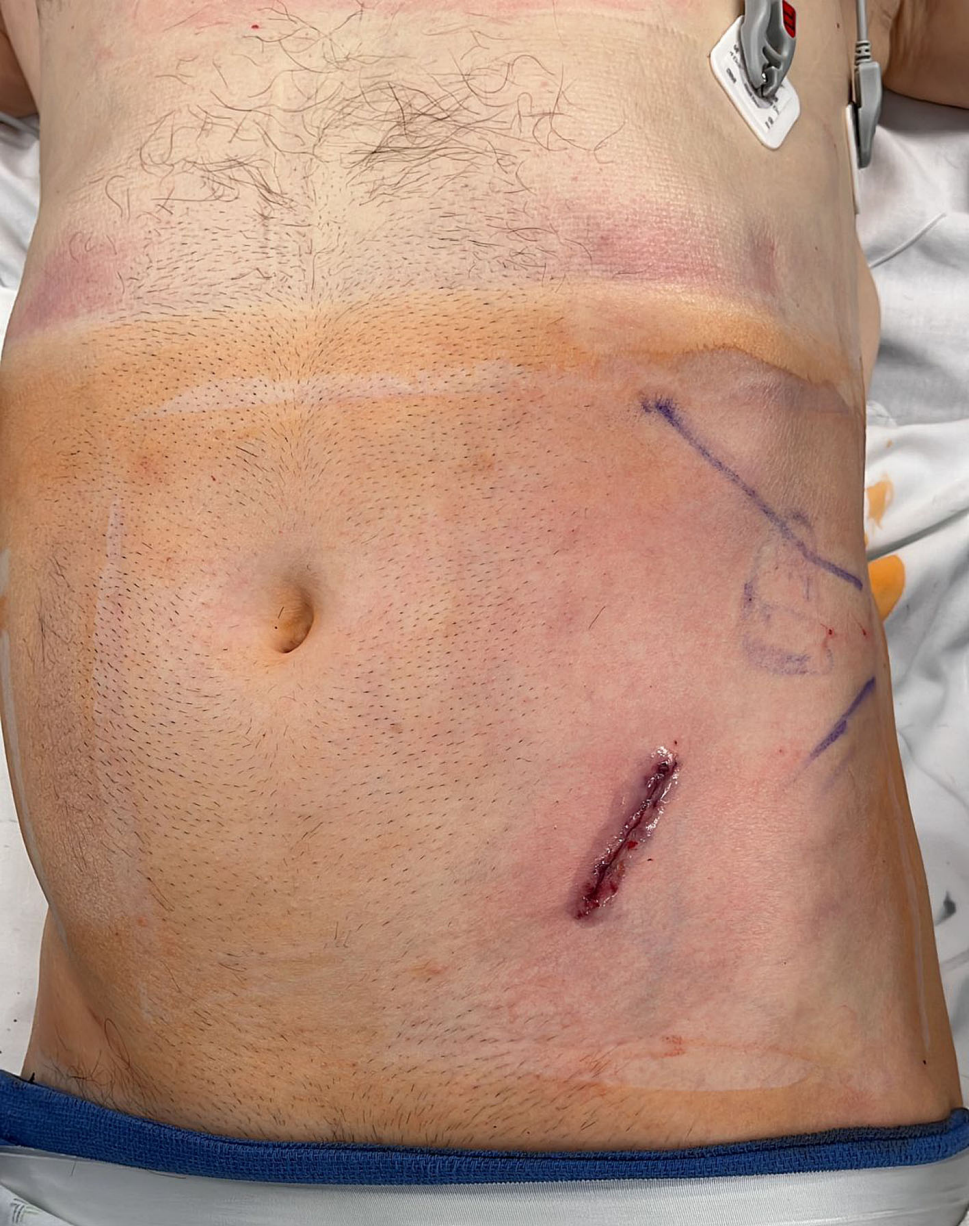
Sample single‐port incision, in this case single‐port‐assisted robotic nephrectomy.

A prospective study was conducted on patients who underwent uro‐oncologic surgery through the SP or MP DaVinci robotic approach performed by three experienced robotic surgeons at the University of Illinois at Chicago. These surgeries included, but were not limited to, prostatectomy, nephrectomy, and cystectomy, which are all common uro‐oncologic procedures. No additional skin incisions were used to introduce an assistant port, which instead entered the surgical field from a separate fascial incision in the side car fashion (see Figure 1 for a sample MP incisions and Figure 2 for SP).

MP and SP incisions were closed with the same technique. The main fascial incision was closed with 0 Vicryl in an interrupted fashion. Subcutaneous tissue was approximated with a 3–0 Vicryl in an interrupted fashion. The skin was then closed with 4–0 Monocryl in a subcuticular fashion. The incisions were dressed with skin glue. Additional fascial incisions for the MP platform larger than 8 mm were closed with the Carter–Thomason closure system.

From April to November 2021, patients were administered the Patient Scar Assessment Questionnaire (PSAQ)—a reliable measure of patient perception of surgical scars. The PSAQ includes five subscales: Appearance, Symptoms, Consciousness, Satisfaction with Appearance, and Satisfaction with Symptoms. Global assessment questions in each category (e.g., overall appearance, overall irritability from symptoms, overall self‐consciousness from the scar, overall satisfaction with the appearance, and overall satisfaction with level of irritation from symptoms) were omitted. In total, 34 questions were asked. Each subscale consists of categorical response options, with scores ranging from 1 to 4. Favourable responses correlate with lower values on the scale and unfavourable responses with higher values.^14^, ^15^

Questionnaires were administered over the phone. Calls were conducted by study members which were not part of the surgical team. Patients were called 20 days post‐surgery and 90 days post‐surgery to allow for evaluation of their satisfaction during the proliferative and maturative stages of their surgical wound healing, respectively.^16^

Prior to conducting the PSAQ, interviewers confirmed patient identification and emphasized the voluntary nature of the survey through the phone. To increase the response rate, each patient was called up to three times to record a response to the survey. The majority of patients answered the questionnaire within the same week of the postoperative time points. However, patients who answered the questionnaire within 1 month of the postoperative time points were also included. Partial responses to the questionnaire were not included in the analysis. Translator services through the University of Illinois Health System were utilized for individuals with a primary language other than English. Patient responses were recorded in a limited‐access, secure document.

## 3 RESULTS

A total of 62 SP and 29 MP uro‐oncologic procedure recipients were selected to participate in this study. Altogether, information from 53 SP (85% response rate) and 24 MP (82% response rate) patients at Postoperative Day 20 and 24 SP (38% response rate) and 13 MP (44% response rate) patients at Postoperative Day 90 was collected. Analyses of differences between the SP and MP groups regarding time from surgical completion to questionnaire completion, pain level, and subscale responses were conducted through *t*‐test.

On Postoperative Day 20, a *t*‐test indicated that there was no significant difference between the time from surgical completion to questionnaire completion (Mean = 24 ± 6 days for the SP group and Mean = 23 ± 7 days for the MP group [*p* = 0.82]). On a scale from 0 to 10, there was no significant difference between the level of pain between the SP group (Mean = 2 ± 2) and the MP group (Mean = 2 ± 2) (*p* = 0.65). The appearance subscale of the PSAQ for the SP group (Mean = 14 ± 3) was significantly lower than that of the MP group (Mean = 17 ± 4) (*p* < 0.001). The symptoms subscale of the PSAQ for the SP group (Mean = 8 ± 2) was significantly lower than that of the MP group (Mean = 10 ± 4) (*p* = 0.012). The consciousness subscale of the PSAQ for the SP group (Mean = 9 ± 2) was significantly lower than that of the MP group (Mean = 11 ± 3) (*p* < 0.001). The satisfaction with appearance subscale of the PSAQ was not significantly different between the SP group (Mean = 12 ± 4) and the MP group (Mean = 13 ± 3) (*p* = 0.32). Similarly, the satisfaction with symptoms subscale of the PSAQ was not significantly different between the SP group (Mean = 8 ± 3) and the MP group (Mean = 9 ± 3) (*p* = 0.47). In addition, a chi‐squared test indicated that SP patients (21%) were significantly less likely to report taking pain medication than MP patients (50%) (*p* = 0.009). Results for Postoperative Day 20 are summarized in Table 1.

**TABLE 1:** Single‐port versus multiport robotic‐assisted procedures at Postoperative Day 20.

Questionnaire metric	Total (*N* = 77)	Multiport (*N* = 24)	Single port (*N* = 53)	*p*‐value
Days since surgery (*SD*)	24 (6)	23 (7)	24 (6)	0.82
Pain score 0 to 10 (*SD*)	2 (2)	2 (2)	2 (2)	0.65
% taking pain medication	30%	50%	21%	**0** **.009**
Appearance (*SD*)	15 (4)	17 (4)	14 (3)	**<0.001**
Symptoms (*SD*)	8 (3)	10 (4)	8 (2)	**0.012**
Consciousness (*SD*)	10 (3)	11 (3)	9 (2)	**<0.001**
Satisfaction with appearance (*SD*)	12 (4)	13 (3)	12 (4)	0.32
Satisfaction with symptoms (*SD*)	8 (3)	9 (3)	8 (3)	0.47

*Note*: Items in bold indicate statistical significant results.

On Postoperative Day 90, a *t*‐test indicated that there was no significant difference between the time from surgical completion to questionnaire completion (Mean = 94 ± 6 days for the SP group and Mean = 94 ± 7 days for the MP group [*p* = 0.76]). On a scale from 0 to 10, there was no significant difference between the level of pain between the SP group (Mean = 0 ± 1) and the MP group (Mean = 0 ± 1) (*p* = 0.59). The appearance subscale of the PSAQ for the SP group (Mean = 12 ± 2) was significantly lower than that of the MP group (Mean = 14 ± 3) (*p* = 0.016). The symptoms subscale of the PSAQ for the SP group (Mean = 7 ± 1) was not significantly different from that of the MP group (Mean = 7 ± 1) (*p* = 0.58). The consciousness subscale of the PSAQ for the SP group (Mean = 8 ± 2) was not significantly different from that of the MP group (Mean = 9 ± 1) (*p* = 0.091). The satisfaction with appearance subscale of the PSAQ was not significantly different between the SP group (Mean = 11 ± 4) and the MP group (Mean = 10 ± 2) (*p* = 0.51). Similarly, the satisfaction with appearance subscale of the PSAQ was not significantly different between the SP group (Mean = 7 ± 2) and the MP group (Mean = 6 ± 1) (*p* = 0.51). On Postoperative Day 90, zero patients from both the SP and MP groups reported taking any pain medication. Results for Postoperative Day 90 are summarized in Table 2. Specific operations performed and their relative frequency are summarized in Table 3.

**TABLE 2:** Single‐port versus multiport robotic‐assisted procedures at Postoperative Day 90.

Questionnaire metric	Total (*N* = 37)	Multiport (*N* = 13)	Single port (*N* = 24)	*p*‐value
Days since surgery (*SD*)	94 (6)	94 (7)	94 (6)	0.76
Pain score 0 to 10 (*SD*)	0 (1)	0 (1)	0 (1)	0.59
% taking pain medication	0	0	0	
Appearance (*SD*)	13 (3)	14 (3)	12 (2)	**0.016**
Symptoms (*SD*)	7 (1)	7 (1)	7 (1)	0.58
Consciousness (*SD*)	8 (2)	9 (1)	8 (2)	0.091
Satisfaction with appearance (*SD*)	10 (3)	10 (2)	11 (4)	0.51
Satisfaction with symptoms (*SD*)	6 (2)	6 (1)	7 (2)	0.29

*Note*: Items in bold indicate statistical significant results.

**TABLE 3:** Main surgical site of surgeries performed with count and frequency.

Site	SP count (%)	MP count (%)
Adrenal	1 (2%)	1 (4%)
Kidney	16 (30%)	5 (22%)
Ureter	5 (9%)	0 (0%)
Bladder	1 (2%)	1 (4%)
Prostate	25 (47%)	14 (61%)
Multiple	5 (9%)	2 (9%)

Abbreviations: MP, multiport; SP, single port.

## 4 DISCUSSION

This prospective study revealed through PSAQ an appreciable cosmetic, symptomatic, and psychometric difference between SP and MP DaVinci robotic approaches among patients receiving uro‐oncological procedures after 20 days. After 90 days, we observed an appreciable cosmetic benefit among patients receiving SP procedures.

PSAQ is a valid and reliable measure of patients' perceptions regarding scarring. It has demonstrated high test reliability across all of its various subscales (intraclass correlation coefficient, 0.74–0.87) and across groups (e.g., receipt of head and neck nevi excision surgery, cardiothoracic surgery, and receipt of incisions by health human volunteers as part of a randomized control trial).^14^, ^15^ The various subscales of the PSAQ can be used independently of each other in order to assess scar changes in specific domains, allowing for the determination of the domain in which the SP system may affect patient perceived outcomes and other domain in which the SP system may not have similar effects.^14^ In this study, we found significant differences in terms of appearance, symptoms, consciousness, and use of pain medication on Postoperative Day 20 between recipients of SP and MP procedures. There were no significant differences in terms of pain score or satisfaction with appearance and symptoms on this day. On Postoperative Day 90, however, there were only significant differences in terms of the appearance subscale.

A 2021 comparative study of surgical scar cosmesis based on an operative approach for radical prostatectomies showed that SP scars without assistant port scars were rated more favourable in terms of appearance when compared with SP scars with assistant ports, multiport scars, and open radical prostatectomy scars.^17^ Our prospective study showed similar results, demonstrating short‐term advantages to the SP approach in the immediate postoperative period in terms of pain, appearance, consciousness, and symptoms. We also showed significant benefit to the SP approach in terms of appearance in the long term. This consensus with the SP technique in the uro‐oncological setting contributes to our understanding of patient preferences in our population of primarily urban‐dwelling economically underserved patients. The patients in our study reported favourable outcomes for the SP technique when compared with the MP technique. While previous studies have demonstrated similar results, this is the first study, to our knowledge, to prospectively compare between Patient Reported Outcomes (PROs) in SP and MP surgical procedures.

However, our study did not consider the impact of patient‐reported favourability on clinical decision‐making. The impact of cosmetic preferences on clinical decision‐making is a topic that warrants further investigation. PROs are an essential element of clinical decision‐making, as they are an important part of determining quality of life and significantly impact therapy outcomes (e.g., medication adherence and adherence to follow‐up).

In addition, the method of data collection in our investigation is another possible limitation. Our study was conducted over the phone and the investigators were not blinded to surgery type, which can cause observer bias. Some may also view phone‐administered interviews as a limitation, but prior studies have found that there is little difference between results from phone‐administered and face‐to‐face interviews.^18^ Furthermore, while we had a high‐response rate (>80%) on Postoperative Day 20, this response rate dropped on Postoperative Day 90. Reasons for the decreased response rate include patient nonresponse to phone calls and unwillingness to further participate. This correlated with a lower number of responses on Day 90, with only 37 (13 MP and 24 SP) responding.

Another limitation is represented by the PSAQ itself, the survey instrument used in this study and developed to assess single linear surgical incisions. Unfortunately, at the time of the completion of this study, no instrument was available to evaluate better the complexity of multiple surgical wounds of the MP platform. However, we believe the results of this study are relevant, demonstrating how a single but longer SP incision can have significant advantages.

Our study did not consider whether the original surgical incision needed to be extended to allow for specimen extraction. Although this could represent a bias, however, we do not believe this to be significant due to the similarity in the frequency of all surgeries that were performed among the two surgical platforms, as indicated in Table 3. Related to this, another significant limitation of our study is the lack of randomization of the surgical platform that was employed to complete the procedure. However, patients were not assigned to undergo either SP or MP based on demographic or clinical characteristics.

Finally, our study was only conducted in a single site with a limited cohort of patients, limiting its generalizability. A future study could be conducted in multiple centres with a larger dataset.

## 5 CONCLUSIONS

Surgical incisions have a significant impact on patient quality of life, impacting sense of well‐being and postoperative pain. Our study was the first to prospectively determine differences in PROs of scars after receiving either SP or MP uro‐oncological procedures. Although this is a limited sample, our findings demonstrate short‐ and long‐term advantages to the SP approach in terms of scar appearance and short‐term advantages to the SP approach in terms of use of pain medication, appearance, and consciousness when compared with the MP approach.

## AUTHOR CONTRIBUTIONS

L. M. and S. C. designed and directed the study. L. M., A. A., A. B., M. P., E. E., A. G., and J. S. surveyed the study participants, completed statistical analysis, and drafted the manuscript. L. M. and S. C. reviewed the final manuscript draft before submission.

## CONFLICT OF INTEREST STATEMENT

There are no conflicts of interest to report.
